# Single-MicroRNA Detection on High-Selectivity Metasurface Fluorescence Biosensors

**DOI:** 10.1021/acsnano.5c15853

**Published:** 2025-10-28

**Authors:** Masanobu Iwanaga

**Affiliations:** Research Center for Electronic and Optical Materials, National Institute for Materials Science (NIMS)^RINGGOLD^ , 1-1 Namiki, Tsukuba 305-0044, Japan

**Keywords:** metasurface, biosensor, microRNA, single-molecule detection, selective biosensing

## Abstract

Next-generation diagnostics is expected to use the abundant data on living bodies and provide sufficiently useful healthcare information. A significant portion of the data are considered to be collected from microRNAs (miRNAs), which play crucial roles in various activities inside the body. Here, we demonstrate single-miRNA detection using metasurface fluorescence (FL) biosensors, which are optimized all-dielectric nanostructured surfaces featuring excellent FL detection capability. Ultimate high-sensitivity discrimination of one miRNA from zero miRNA is achieved at the subattomolar level by employing optimized reverse transcription (RT) of miRNAs, polymerase chain reaction (PCR) suppressing false reactions, and highly efficient and target-selective FL detection of the miRNA amplicons on the metasurface biosensors using appropriately designed oligo DNA probes. This degree of precision has never been obtained using any other technique, such as digital PCR, which is currently one of the most efficient techniques. Furthermore, we demonstrate the specific detection of a cancer-correlated miRNA that is deeply mixed with another miRNA. We also examine and discuss other methods that possibly work for miRNA detection at femtomolar or lower concentrations, such as chromatography and different amplification methods, including handy one-step RT-PCR.

## Graphical abstract

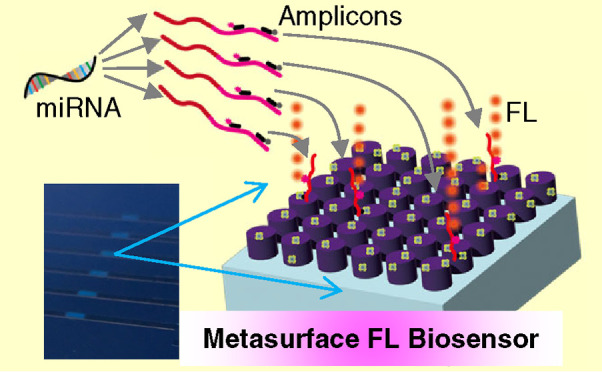

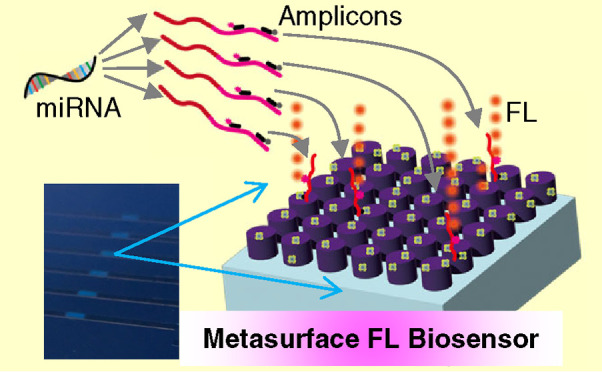

MicroRNAs (miRNAs), comprising approximately 20 bases, are currently crucial biosensing targets because of their involvement in diverse activities in living bodies, indicating disease-related signatures, even in the early stages. Substantial volumes of the information on miRNAs have been accumulated in databases, one of which is an open Web site.^1,2^ Many miRNAs are most likely correlated with cancers, and a part of them is considered to serve as markers at the early stage diagnostics^3–7^ and noninvasive examinations,^8^ which are being pursued extensively and have not yet been established as medical examinations. For example, hsa-miR-15a-5p and hsa-miR-143-3p were suggested to be associated with several diseases such as hepatocellular carcinoma, colorectal cancer, and pancreatic cancer.^1^

Typical procedures to obtain miRNA involve several steps, as illustrated in Figure 1A. From the sampling of a biopsy to the final collection of the miRNA, substantial effort is required. Various trials using magnetic beads, porous materials, and so on are commercially underway to improve these processes. These pretreatments for collecting miRNAs are out of the scope of this study.

**Figure 1:**
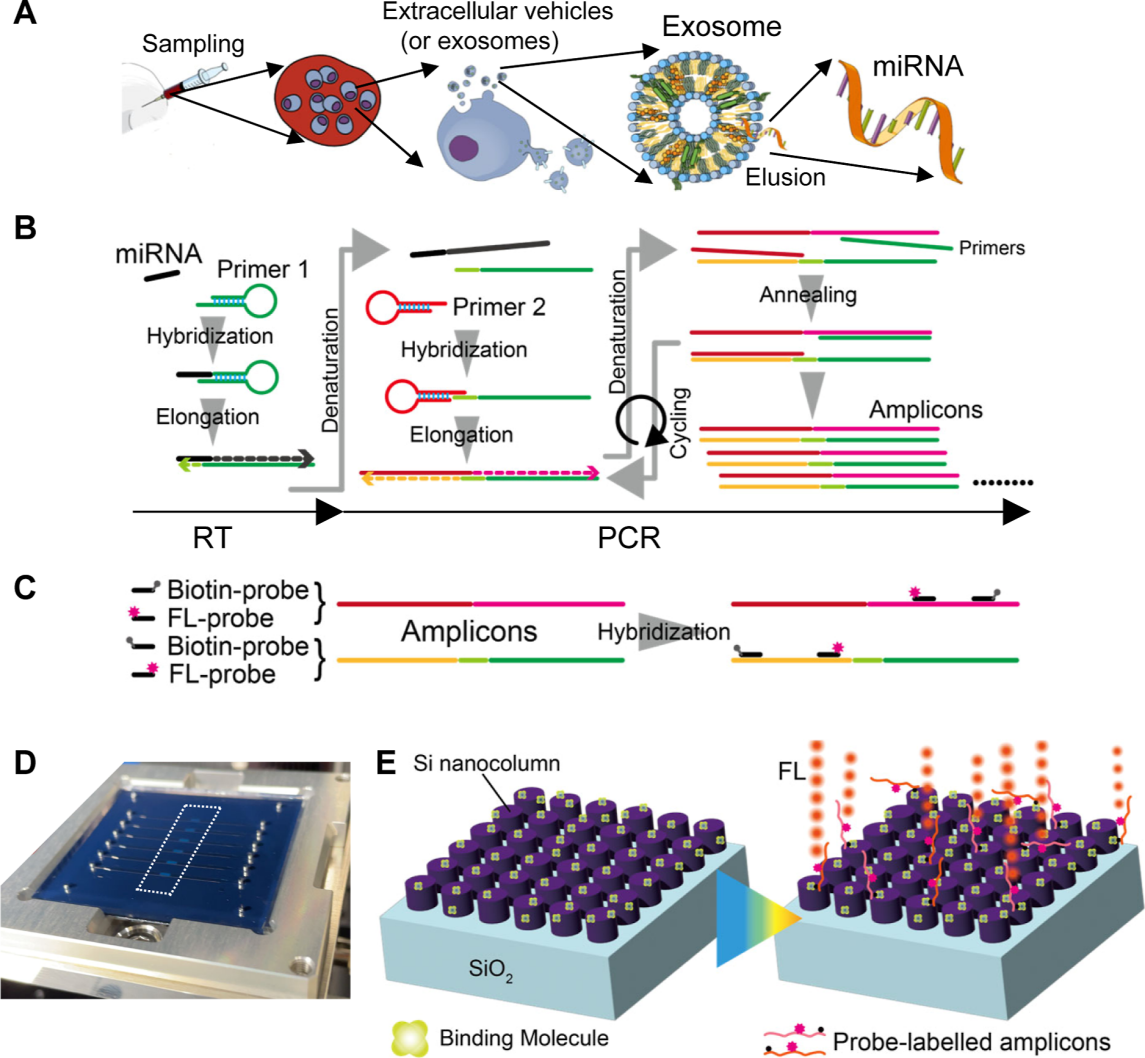
Schematics of microRNA (miRNA) collection, amplification of miRNA, hybridization with probes, and metasurface fluorescence (FL) biosensors. (A) A typical way of sampling of miRNA. (B) Two-step reverse transcription (RT) polymerase chain reaction (PCR) to amplify the target miRNA(s) and yield amplicons. (C) Hybridization of biotin- and FL-probes with the amplicons. (D) Photo of a six-channel metasurface FL biosensor chip with lateral dimension of 45 mm × 45 mm, which is set in a holder. (E) Illustration of FL detection on the metasurface biosensor, comprising a periodic array of silicon nanocolumns. After immobilization of binding molecules, the target amplicons with biotin labels are captured and exhibit enhanced FL emission.

Several methods were pursued to detect collected miRNAs with a high precision. RT of miRNAs to complementary DNAs (cDNAs) and PCR of the cDNA are currently the most common technique. In conventional quantitative PCR (qPCR), fluorescence (FL) probes are added to amplicons, and FL signals are detected as the amplification progresses. In the early stage of RT-PCR trials, it was reported that ten types of miRNAs were tested, one of them showed a limit of detection (LOD) of 0.2 femtomolar (fM), six showed an LOD of 2 fM, and three showed an LOD of 20 fM;^9^ thus, the typical LOD was approximately 2 fM. Although trials to attain lower LODs in miRNA detection are rare, RT-PCR for long RNA of hundreds of bases was extensively tested during the pandemic due to COVID-19. The RT-qPCR for the virus RNA was reported to have an LOD of 30–50 copies/test.^10^ An improved PCR technique is digital PCR (dPCR), which uses fractionation plates and implements elaborate statistical analysis to obtain an improved LOD.^11–13^ Further trials to improve the LOD are conducted using droplets for PCR, called droplet dPCR (ddPCR). The best performance was claimed to be 5 copies/test.^14^

Apart from the PCR techniques, another approach for miRNA detection was based on loop-mediated isothermal amplification (LAMP);^15^ as the best performance, detection of 6 copies/test was claimed. As is widely known, LAMP is more elaborate than PCR,^16,17^ requiring four types of primers, whereas PCR uses only two types. Although attempts have been made to attain higher sensitivity for DNA using LAMP,^18–20^ single DNA detection has not been succeeded so far. As a simpler and more improved procedure compared with the previous study,^15^ the RT-PCR for miRNAs to use two types of primers (primers 1 and 2) was adopted, as shown in Figure 1B.

To pursue extreme high-precision capability enabling single-miRNA detection, metasurface FL biosensors^21,22^ are a candidate because they are successfully detected in single cell-free DNA (cfDNA),^23^ which is a short fragment of the full-length gene. Figure 1D shows a photograph of a metasurface FL biosensor chip, which is placed in a holder and comprises a self-absorbed pair of a metasurface substrate and a transparent microfluidic (MF) chip made of polydimethylsiloxane (PDMS); six areas (blue in the dotted-line box) aligned in the vertical direction are metasurfaces. Manipulation of liquid flows in the MF channels and FL measurement on the metasurface biosensors are automated.^23^ Figure 1E illustrates a standard molecular configuration to detect FL signals on the metasurface FL biosensors. Initially, binding molecules of cysteine-streptavidin (Cys-SA) are immobilized; subsequently, biotinylated amplicons are effectively captured via biotin–streptavidin binding; finally, LED-light excitation induces the FL signals. The metasurface consists of a periodic silicon-nanocolumn array of 200 nm height on a silicon dioxide layer. The metasurface biosensors exhibit outstanding FL-intensity enhancement^24^ among numerous trials for metasurface nanofabrication,^25^ functioning as efficient FL biosensors.^23,26–30^ Further details of the FL detection, the metasurface structures, and the fabrication process are described in the Experimental Section.

In this study, we aimed at demonstrating single-miRNA detection employing metasurface FL biosensors. For conducting explicit quantitative experiments, we used synthesized single-strand miRNAs as detection targets. As representative miRNAs among numerous miRNAs known to date,^1,2^ hsa-miR-15a-5p and hsa-miR-143-3p are here set to be the targets. Although miRNAs exist in cells and biopsy samples in reality, the concentrations are undetermined, which prevents us from their quantitative evaluation in practice. After extensive explorations of protocols in two-step RT-PCR and FL detection on the metasurface biosensors, we achieved single-miRNA detection and substantiated robust detection under mixed miRNA conditions.

## Results and Discussion

### Single miRNA Detection

Figure 2A shows a representative set of miRNA-detection FL images of the metasurface biosensors, acquired by a noncooling CCD camera; the colored areas correspond to the metasurfaces; clearly, areas outside the metasurfaces are dark, suggesting the FL background due to nonspecific absorptions is very low. The target miRNA was hsa-miR-15a-5p, the sequence of which is listed in the Experimental Section. The target concentrations were in an attomolar (aM) range from 500 attomolar (aM) to 0 molar (M).

**Figure 2:**
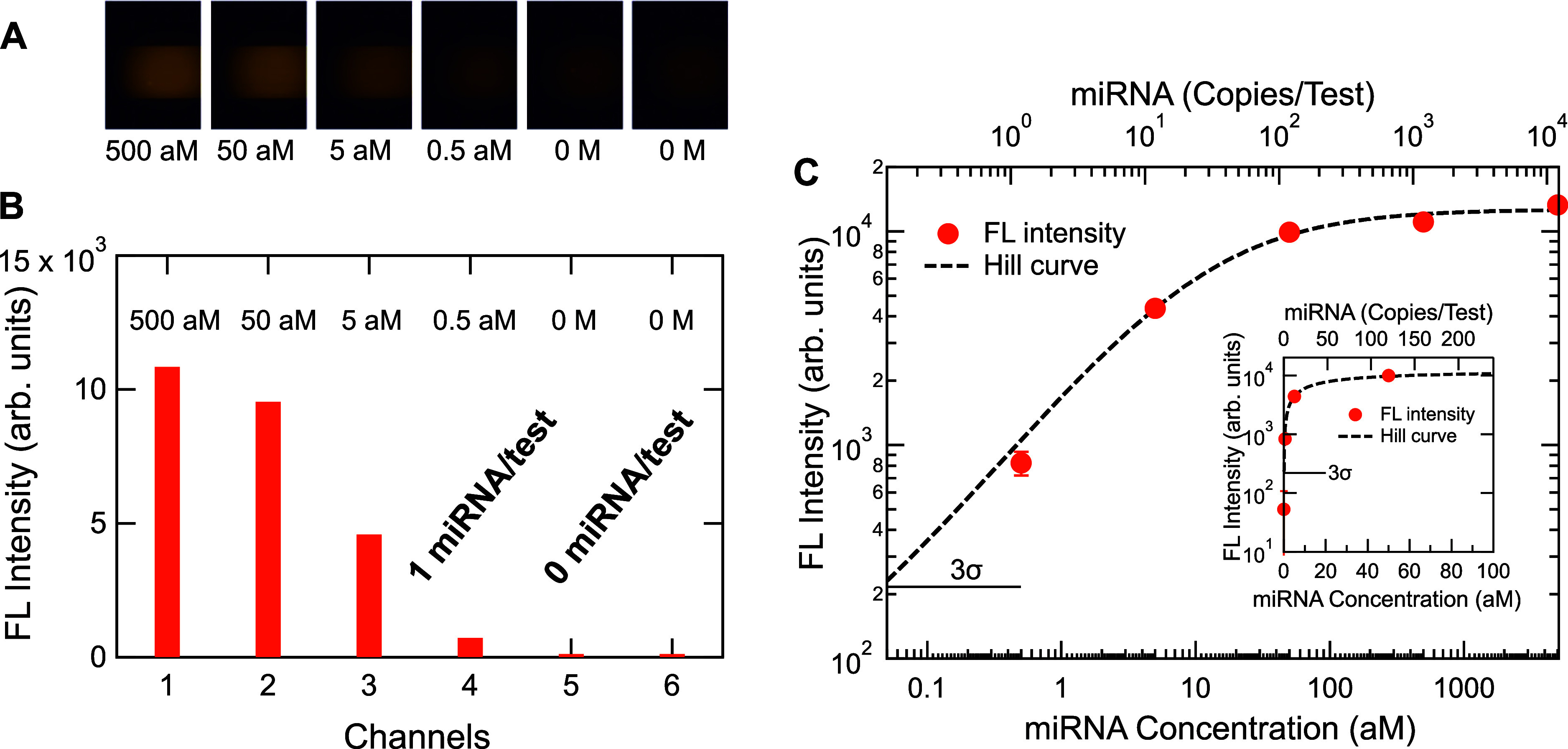
A representative result of miRNA detection on the metasurface FL biosensors. (A) FL images of target miRNA, hsa-miR-15a-5p, concentrations from 500 attomolar (aM) to 0 molar (M), displayed from left to right. (B) Net FL intensity evaluated from the FL images in (A). (C) Measured FL intensity versus target miRNA concentration in aM (bottom) or copies/test (top), plotted on a log–log scale. The fitted Hill curve defined in eq 1 is shown with a dashed curve. The horizontal bar indicates the 3σ line from the zero-concentration data (σ: standard deviation). Inset provides a magnified view at low concentrations of 0–100 aM on a semilinear scale, including the data point at 0 M.

In Figure 2B, we show the quantitative net FL intensities (orange bars), which were evaluated by subtracting the FL intensities shown in Figure 2A from the background intensities measured immediately after the binding-molecule immobilization. The miRNA concentration of 0.5 aM corresponds to 1 miRNA/test in the experiment. The miRNAs of 0 M represent negative control. Evidently, the FL intensity at 1 miRNA/test is distinct from those at 0 miRNA/test.

In Figure 2C, we present experimental data (orange dots with error bars) in an extremely low-concentration range from 5000 to 0.5 aM, which corresponds to a range from 11,800 to 1.18 copies/test. Practically, 1.18 copies/test is equivalent to a single miRNA test. In this measurement, we implemented the detection of miRNAs at extremely low concentrations with conducting 45-cycle PCR after the RT reaction. The detailed experimental conditions are provided in the Experimental Section. The measured data plotted on a log–log scale were fitted using a Hill curve (dashed black curve), defined in eq 1; the profile was described using the Hill equation because it quantifies acceptor–analyte coupling products under equilibrium conditions;^31,32^ indeed, the capture reaction of biotin-labeled amplicons on the metasurface biosensors in microfluidic channels under low flow rate occurs under an equilibrium condition:

1 $$y=y_{0}+\left(S-y_{0}\right)\frac{x^{n}}{x^{n}+K_{D}^{n}}$$

where *y* is the FL intensity; *y*_0_ is the FL intensity at zero concentration, representing with negative control; *x* is the concentration of miRNA; *S* is the saturation value; *K*_D_ is the dissociation factor; and *n* is an index representing cooperative/anticooperative reaction. By fitting the experimental data in Figure 2C, a set of parameters was determined such that *y*_0_ = 52.7, *S* = 12,689, *K*_D_ = 11.7, and *n* = 0.778. We note that the value of *y*_0_ was experimentally determined using the averaged FL intensities at 0 M, which was a negative control; the value was less than 0.1% in the detection range of the CCD camera. A short horizontal bar indicates the 3σ line (σ: standard deviation) from the negative control. Furthermore, the inset presents the data and Hill curve on a semilinear scale at 0–100 aM, including the data at 0 M. Obviously, the FL intensity of 1 copy/test is above the 3σ line, which statistically guarantees that the single-miRNA signal is discriminated from the zero-miRNA signal. The value of *n* < 1 implies that the binding reaction between the immobilized Cys-SA and the miRNA amplicons with the biotin probes was anticooperative, which was often observed in configurations to use low-concentration analytes.^23,27,29,33^ Owing to the large PCR cycles, the dissociation factor *K*_D_, which gives the center concentration of the S-shape Hill curve on the linear scale of the *y* axis, was reduced to approximately 12 aM. We remark that the Hill equation is equivalent to the 4-parameter equation,^26^ which is frequently used to analyze concentration-dependent biosensing data.

In Figure 3, comprehensive detection results of miRNA, hsa-miR-143-3p, are shown. Figure 3A shows the miRNA detection in a wide range of concentrations from 10 picomolar (pM) to 50 aM; the concentrations are represented on a semilog scale. To cover this wide range more than six orders of concentrations, different PCR cycles were conducted; open black squares, closed green triangles, and closed red circles correspond to 35, 40, and 45 PCR cycles, respectively. Dashed curves are fitted to Hill curves (eq 1) for each measured set. The parameters *K*_D_ were 480.3, 59.5, and 6.7 fM for the PCR cycles of 35, 40, and 45, respectively, indicating that the detection range of concentrations can be changed by varying the cycles. Although the 5-cycle increase, in principle, results in 32-fold amplification, the *K*_D_ values mean less than 10-fold amplification. Thus, the PCR process deviated from the ideal amplification due to the combination of the miRNA, primers, and reagent kit.

**Figure 3:**
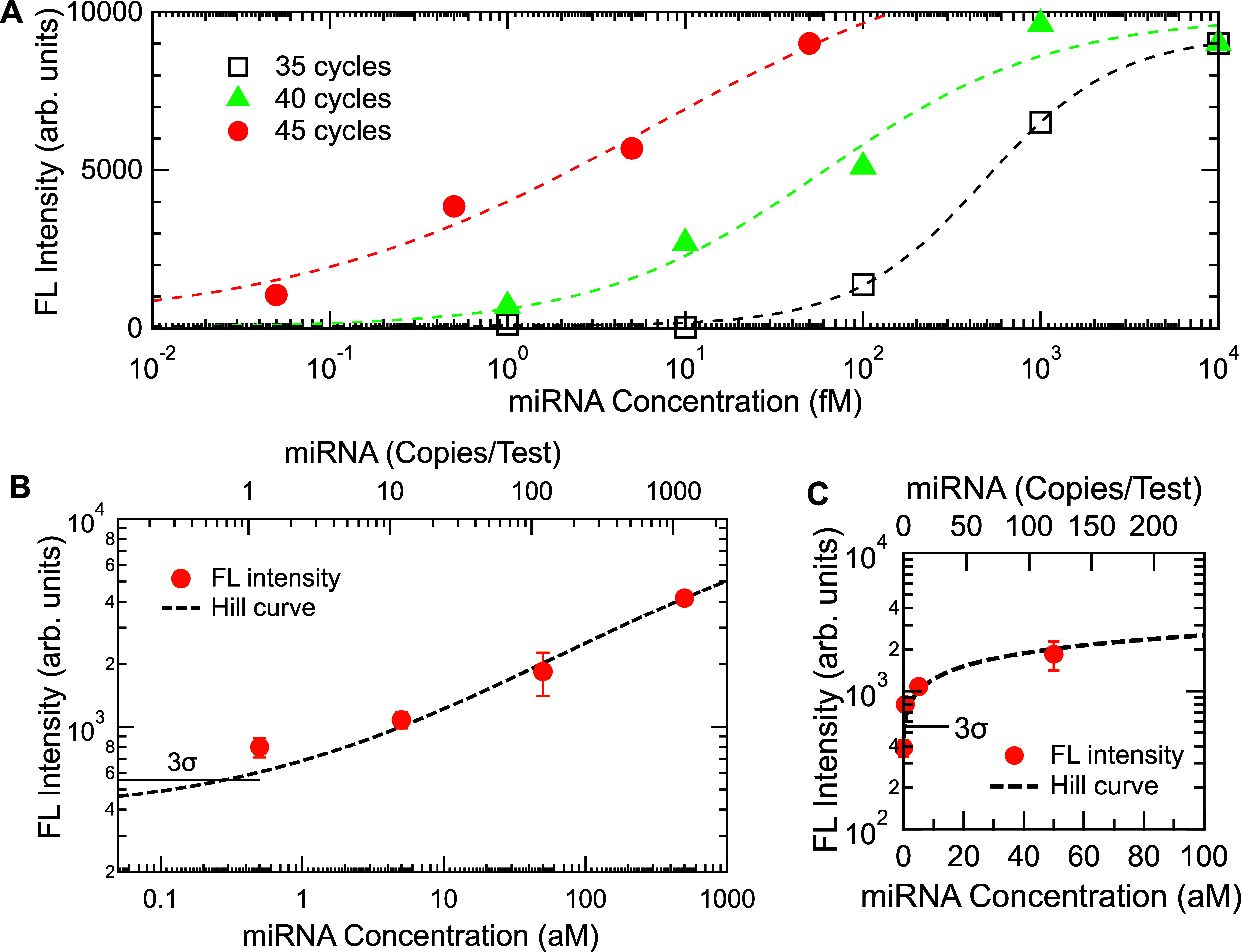
(A) A series of miRNA, hsa-miR-143-3p, detections in a wide range from 10,000 fM to 50 aM on a semilog scale. Data shown with black square, green triangles, and red circles were measured after going through 35, 40, and 45 PCR cycles, respectively. For clarity, the FL intensities at the highest concentration in the three series are set to be equal. All the dashed curves are Hill curves, defined in eq 1. (B,C) Data plots regarding single-miRNA detection, shown on log–log and semilinear scales, respectively. Measured data in a range from 500 to 0.5 aM are presented with orange dots with error bars, shown together with the fitted Hill curve (dashed black), which is defined in eq 1. The horizontal bar indicates the 3σ line from the zero-concentration data.

We remark that the FL-intensity range in Figure 3A, which is at most 4 orders of magnitude, is determined primarily not by the detection capability of metasurface biosensors but by the set of reagents and primers in the RT-PCR. This is understood as follows: the metasurface biosensors comprise a square array of 300 nm periodicity, thereby having 1.11 × 10^7^ Si nanocolumns/mm^2^; each Si nanocolumn functions as an FL-enhancing optical resonator;^24^ consequently, when FL-labeled analytes are ideally immobilized on all the nanocolumns, more than 10^7^ sites emit FL; thus, the intrinsic detection range (or dynamic range) of the metasurface biosensors is, in principle, more than 7 orders of magnitude, which is considered to be limited in Figure 3A by the immobilization efficiency and actual range of analyte concentrations.

Figure 3B,C shows the detection profile of miRNA of hsa-miR143-3p on the log–log and semilinear scales, respectively, measured after implementing 50 PCR cycles; orange dots with error bars indicate the measured FL intensities, a dashed black curve fits the FL intensities using eq 1, and a horizontal bar denotes the 3σ line from the FL-intensity level at the concentration of 0 M. The FL signal at 1 copy/test (i.e., 0.5 aM) is definitely discriminated from the FL signal at 0 copy/test, thus substantiating the single-miRNA detection. In fitting the experimental data of Figure 3B using eq 1, a set of parameters were determined such that *y*_0_ = 386.9, *S* = 12,200, *K*_D_ = 2556.2, and *n* = 0.465. We note that the value of *y*_0_ was experimentally determined similarly to that in Figure 2C. The factor *K*_*D*_ remained at a large value of 2556.2 aM even under 50 cycles of PCR amplification, implying that the RT-PCR is less efficient for hsa-miR-143-3p than for hsa-miR-15a-5p, despite the similarity of their primer designs. Generally, the efficiency of RT-PCR depends on the target miRNAs and primers.

### Selective Detection of miRNAs

Figure 4 presents a series of specific detection results of hsa-miR-15a-5p miRNA as a 3D bar graph. In this experiment, the target miRNA was mixed with another miRNA, hsa-miR-143-3p. We started solutions containing the target and counter miRNAs and used primers only for the target in the two-step RT-PCR. The target concentrations were varied from 50 fM to 50 aM, whereas the concentrations of the mixed miRNA were set to 5 fM, 500 aM, or 50 aM. The PCR cycles were set to 45 cycles. Clearly, the three series of target miRNAs were detected regardless of the mixed-miRNA concentrations. In particular, the target miRNAs were detected even when the concentration of the mixed miRNA at 5 fM was 100-fold higher than that of the target miRNA at 50 aM. These results declare that the present scheme for miRNA detection, which combines two-step RT-PCR with the metasurface FL biosensors, is a robust method.

**Figure 4:**
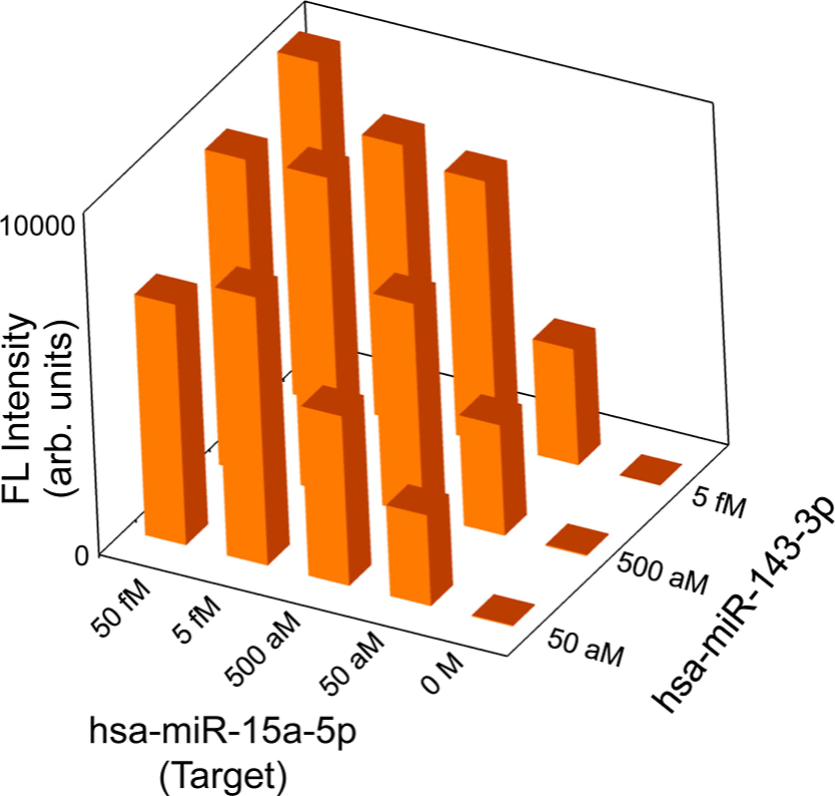
3D-bar presentation of specific detection of the target miRNA, hsa-miR-15a-5p, which was mixed with another miRNA, hsa-miR-143-3p. The probe set was used only for the target. Selective detection of the target miRNA was obtained, even when the target miRNA was 100-fold lower in the concentration than the mixed miRNA.

### Discussion on Other Detection Techniques

#### One-Step RT-PCR

We performed one- and two-step RT-PCRs using common reagents, such as polymerases, as described in the Experimental Section. As a result, we found that the one-step RT-PCR was inferior to the two-step RT-PCR because the one-step process frequently yielded unstable reactions and sometimes false-positive reactions. The background FL level sometimes rose and could not be completely suppressed, even after adjustment of the one-step conditions. Therefore, we adopted two-step RT-PCR in this study.

One-step real-time RT-PCR is a conventionally available, handy technique. We examined this, as described in the results in the Supporting Information (Section S1). Summing up the results, it turned out that the one-step real-time RT-PCR was an unreliable technique to detect the present miRNAs at concentrations of 1 pM or less because of the poor or false-positive reactions irrespective of the miRNA concentrations (Figure S1).

In terms of short-time and point-of-care PCR, infrared-light-heating metasurfaces were recently reported.^34^ The rapid heating at 16.6 K/s and cooling at 7.7 K/s may make PCR cycling more feasible in a compact setup with low-power consumption.

#### LAMP

One of the lowest concentrations detected was reported using RT-LAMP.^15^ We followed the primer designs and LAMP procedures to detect miRNAs in this study. Eventually, we could not reproduce the main claim of 6 copies/test detection;^15^ only 1686 copies/test (or 2 fM) and higher concentrations were detectable. Our typical RT-LAMP results are shown in the Supporting Information (Figure S2) together with the experimental details. As shown in Figure 1B, we substantially simplified the elaborate RT-LAMP that requires the four types of primers, leading to single-miRNA detection in this study.

#### Chromatography for RT-PCR Products

After RT-PCR, chromatography is a possible technique to detect the amplicons. We tested it using an instrument that uses MF chips to obtain improved chromatography results (Labchip GX Touch 24, Revvity, Waltham, MA, USA) and found that the signals corresponding to the amplicons appeared, irrespective of the target miRNA concentrations. The experimental results are shown in the Supporting Information (Figures S3 and S4) together with detailed descriptions. These results most likely come from the fact that the instrument cannot discriminate the genuine amplicons from unintended amplified DNAs; indeed, the RT-PCR products tend to include a smear. The chromatography is designed to analyze the multicomponent products based only on the mass. In contrast, the metasurface FL biosensors incorporate biotin- and FL-probes for the RT-PCR products, as shown in Figure 1C, and ensure highly selective detection of the genuine amplicons, as shown in Figures 2–4.

#### Other Reported Techniques

At the end of the discussion, we refer to two techniques for miRNA detection reported so far, which took different approaches from those described above. They detected miRNA without using nucleic-acid amplification techniques.

One technique used gold triangular nanoparticles,^35^ which have local surface plasmon resonances (LSPRs) and show a resonant wavelength shift in accordance with the amount of captured miRNAs. The resonant shift is the principle of LSPR-based detection. The LOD was claimed to be 1 fM. However, the miRNA concentrations were varied by six orders (or from 1 nanomolar (nM) to 1 fM), while the change of resonance shift was at most 2%; therefore, the dynamic range of the detection signals was very narrow, and the detected signals at different concentrations overlapped largely to each other. Therefore, the resonance-shift technique is a nonquantitative method, which is a definite drawback. Another study using gold nanospheres^36^ was stimulated by a similar motive to that of the previous study^35^ and concluded that the LOD was 1 pM. These reports suggest that the resonance-shift techniques are unlikely for to be efficient at low concentrations below 1 fM.

The other technique was based on chemical luminescence (CL).^37^ Target miRNAs were first incubated together with biotin-labeled counter DNA probes and anti-DNA/RNA antibodies that were immobilized in advance on magnetic beads; after washing the DNA probes that were not hybridized with the miRNA, streptavidin-labeled luciferase was added as the CL source and incubated; finally, the luciferase unbounded to the DNA probes was rinsed; then, the CL was measured using a commercial instrument.^37^ A range of miRNA concentrations from 1 nM to 10 fM was evaluated, and the LOD was determined to be 6.3 fM, suggesting that this technique is suitable for miRNAs at 10 fM and higher concentrations.

## Conclusions

We have demonstrated single-miRNA detection, which was obtained by going through the two-step RT-PCR suppressing false-positive reactions and the highly selective, efficient FL detection on the metasurface biosensors. In particular, the single miRNA was discriminated from zero, which is an ultimate sensitivity that has never been attained. Furthermore, robust detection under mixed miRNA conditions was tested and substantiated. Several other techniques to detect miRNAs were also discussed together with our experimental examinations. Thus, we reached an ultimate goal in biosensing technology for promising target miRNAs, making the best use of the metasurface FL biosensors with both high sensitivity and selectivity.

## Experimental Section

### miRNA and Probes

Table 1 lists the sequences of the miRNAs, primers, and probes used in this study, which were synthesized and purified through high-performance liquid chromatography (Eurofin Genomics, Tokyo, Japan). The roles of the primers and probes are illustrated in Figure 1B,C. The primers for miRNAs generally need sequences of more than 60 bases because the miRNAs are approximately 20 bases. In contrast, ordinary PCR for DNAs, which are mostly 100 bases or longer, allows the primers to be short (∼20 bases). Considering these points, we adapted a primer design from the study on LAMP for miRNA,^15^ where four types of primers were prepared to conduct LAMP for one type of miRNA. The primers were conceived to reduce false reactions by introducing the loop structure, as seen in Figure 1B. Our results following this LAMP technique are described in the Discussion section.

**Table 1:** Sequences of miRNA, Primers, and Probes, Which Are Displayed from 5′-End (Left) to 3′-End (Right)^c^

species	sequence
hsa-miR-15a-5p	UAGCAGCACAUAAUGGUUUGUG
primer 1^a^	GCTGACGACTCCTTTTGTTGTCTGG-
	AAGTGTGACGCGATTTAGGACTCGT-
	CAGCTTTTTCACAAACCATT
primer 2^b^	TAGCAGCACTGACTTTGTAATAGG-
	ACTGTCCGCCGCACTTTGTCAGTG-
	CTGCTATTTTTAGCAGCACAT
biotin-probe 1	[Bio]AAATCTGGAAGTGTGACGCGAT
biotin-probe 2	[Bio]AAATGTAATAGGACTGTCCGCC
FL-probe 1	CAGCTTTTTCACAAACCAAAT[TAM]
FL-probe 2	CTGCTATTTTTAGCAGCATTA[TAM]
hsa-miR-143-3p	UGAGAUGAAGCACUGUAGCUC
primer 1^a^	GCTGACGACTCCTTTTGTTGTCTGG-
	AAGTGTGACGCGATTTAGGACTCGT-
	CAGCTTTTTGAGCTACAGT
primer 2^b^	CACTGACTTTGTAATAGGACTGTCC-
	GCCGCACTTTGTCAGTGCTGCTATT-
	TTTTGAGATGAGATGAAGC
biotin-probe 1	[Bio]AAATCTGGAAGTGTGACGCGAT
biotin-probe 2	[Bio]AAATGTAATAGGACTGTCCGCC
FL-probe 1	GGACTCGTCAGCTTTTTGTTT[TAM]
FL-probe 2	GTGCTGCTATTTTTTGATAT[TAM]

a Primer for RT reaction.

b Primer for PCR.

c Symbols [Bio] and [TAM] denote biotin and FL-molecule TAMRA,^38^ respectively.

Primer 1 in Table 1 contributed to the RT reaction, designed to hybridize with 10 bases of the target miRNA and produce cDNA. In the design, accidental matching ratio to other miRNAs was suppressed down to 9.77 × 10^–7^. Primer 2 worked in the PCR, hybridizing with 11 bases of the elongated primer 1. Both primers were thus involved in the amplification procedure, as illustrated in Figure 1B.

### RT-PCR

The RT reaction in this study was conducted at 37 °C for 10 min using M-MLV reverse transcriptase (28025013, Thermo Fisher Scientific, Waltham, MA, USA). The PCR reaction was carried out using a KAPA2G Fast PCR kit (KK5500, Roche, Basel, Switzerland); the protocol was implemented using a thermal cycler, such as hot start at 95 °C for 3 min, thermal cycles of (95 °C for 10 s → 45/50 °C for 15/5 s → 72 °C for 15 s) × *N* cycles, and final elongation at 72 °C for 1 min, in which the annealing temperature was set to 45 and 50 °C for hsa-miR-15a-5p and hsa-miR143-3p, respectively, and the cycle number *N* was set in a range from 35 to 50 in each experiment. In the two-step RT-PCR, the RT reaction was conducted first; next, primer 2 and PCR reagents were added to the RT-reaction solution; finally, PCR was conducted, as noted above.

For the two-step RT-PCR, reaction solutions were prepared as follows. The RT-reaction solution of total 10 μL per test contained 2 μL of 20 pmol primer 1, 0.25 μL of 50-unit M-MLV reverse transcriptase, 2 μL of buffer for M-MLV, 1 μL of each 10 nmol dNTP mixture, 4 μL of target miRNA diluted using nuclease-free distillated water (314-09291, Nippon Gene, Tokyo, Japan) with 1 unit/μL RNase inhibitor (0317L, New England Biolabs, Ipswich, MA, USA), and 0.75 μL of RNase-free water with the RNase inhibitor for adjustment of the total amount. The PCR reaction solutions contained the 10 μL RT reaction solution and 15 μL of solution consisting of 2 μL of 20 pmol primer 2, 0.6 μL of 3-unit PCR-polymerase KAPA2G, 5 μL of buffer for the KAPA2G, 1 μL of dNTP mixture associated with the KAPA2G, and 6.4 μL of adjusting nuclease-free water.

After these reactions, the amplicons were hybridized with biotin- and FL-probes, as shown in Figure 1C. In both cases of hsa-miR-15a-5p and hsa-miR-143-3p, the hybridization condition was set to be 95 °C for 3 min → 45 °C for 30 min. As shown in Figure 1C, the probes were designed to couple with the elongated sequences in the RT-PCR, suppressing an increase in unnecessary background FL.

For the one-step RT-PCR, all the primers and reagents were mixed first, being adjusted to a total of 25 μL comprising the 20 μL mixture and 5 μL target miRNA; then, they went through the thermal process without pausing. After this thermal cycling, the probe hybridization was implemented similarly to that in the two-step case.

### Metasurface FL Biosensors

The all-dielectric metasurface biosensors in this study were fabricated using silicon-on-insulator (SOI) wafers composed of the top SOI layer, middle buried-oxide SiO_2_ layer of 375 nm thickness, and bottom Si wafer of 675 μm thickness. The top SOI layer was selectively fabricated through electron-beam lithography for on-top negative resist (NEB-22A, Sumitomo Chemical, Tokyo, Japan) and selective deep reactive ion etching (RIE) only for the SOI layer. The metasurfaces were designed to have 300 nm periodicity and 220 nm diameter of nanocolumns (Figure 1E), being almost faithfully realized, as reported previously.^26,39^ As was examined previously,^24^ the metasurfaces have prominent, almost optimal capability for FL-intensity enhancement at wavelengths of 560–600 nm, where the FL-probe TAMRA (Table 1) emits FL. The large FL-enhancing capability reaching 1000-fold in comparison with a reference flat Si substrate was attained with optimizing the total process from photoexcitation to FL emission.^24^ The analyses and consideration for the whole photoexcited dynamics on metasurfaces were detailed previously.^21,24,40,41^

Slight nanofabrication deviation from the design hardly affected the measured FL signals on the metasurface biosensors; indeed, we used the metasurfaces of diameters of 210–220 nm in this study; the diameters varied from substrate to substrate, and a typical variation on a substrate was 2–3 nm, being suppressed fairly well. Thus, the FL data coming from the same sample were quite uniform on a substrate. The height of Si nanocolumns was determined by the height of the SOI layer (200 ± 5 nm) because the deep RIE etched only the SOI layer without the resist coat. Importantly in practice, the metasurface substrates were reused frequently after washing in piranha solution, which did not induce any detectable damage on the Si nanocolumns forming the metasurfaces.

The MF chips were made of transparent PDMS, which have six channels in accordance with the number of metasurface areas (Figure 1D), which were typically 2.1 × 0.7 mm in lateral size. The height of each MF channel was set to 30 μm, and the total thickness of the MF chips was 2 mm. The MF chips were commercially produced according to our design. Using the MF chips, small volumes (e.g., 50 μL) of liquid samples can be manipulated at low flow rates (e.g., 10 μL/min), which increases FL-measurement reproducibility. Furthermore, each MF channel is isolated, thereby reducing contamination that could happen in handling the amplified products.

The binding molecules, Cys-SA, were flowed at 20 μg/mL, which were diluted using phosphate-buffered saline of pH 7.4, and immobilized onto the metasurfaces (Figure 1E). Owing to the highly efficient biotin–streptavidin coupling, the miRNA amplicons were captured selectively and efficiently on the metasurfaces. The immobilization and capture performance of Cys-SA was explicitly confirmed in the reference experiment in the Supporting Information (Section S4).

## Supplementary Material



## Notes

Experimental results and details of the one-step real-time RT-PCR, LAMP, chromatography, and reference experiment on Cys-SA (PDF)
