# Supplementary material for: Single-MicroRNA Detection on High-Selectivity Metasurface Fluorescence Biosensors
Source: ACS Nano. 2025 Oct 28;19(44):38841–8. doi: 10.1021/acsnano.5c15853 (PMC12613836; doi:10.1021/acsnano.5c15853)
Supplement: Supplementary file 1 [file nn5c15853_si_001.pdf]

Supporting Information: Single-MicroRNA Detection on High-Selectivity Metasurface Fluorescence Biosensors

Masanobu Iwanaga*

Research Center for Electronic and Optical Materials, National Institute for Materials Science (NIMS), 1-1 Namiki, Tsukuba 305-0044, Japan

E-mail: iwanaga.masanobu@nims.go.jp

Note: Abbreviations appeared in the text are used without definitions in this Supporting Information.

S1. One-Step Real-Time RT-PCR

One-step real-time RT-PCR is known as a handy technique to detect RNAs. To our knowledge, very high-precision miRNA detection using one-step real-time RT-PCR has not been reported. We tested a commercial one-step real-time RT-PCR kit (BC-GFQPMM02, GeneFields, South Korea) using the target miRNAs and primers described in the text (Table 1). Figure S1A,B shows a typical result for the miRNAs, hsa-miR-15a-5p and hsa-miR-143-3p, respectively.

The kit runs on a rapid real-time PCR system (GF-Q150, GeneFields). The detection protocol was set as follows, following recommended conditions in the kit. RT was implemented at 50 °C for 5 min; succeedingly, thermal activation of PCR polymerase was set at 95 °C for 30 s; PCR cycles were (95 °C, 5 s → 50/45 °C, 5 s → 72 °C, 10 s) × 50 cycles, where the annealing temperatures

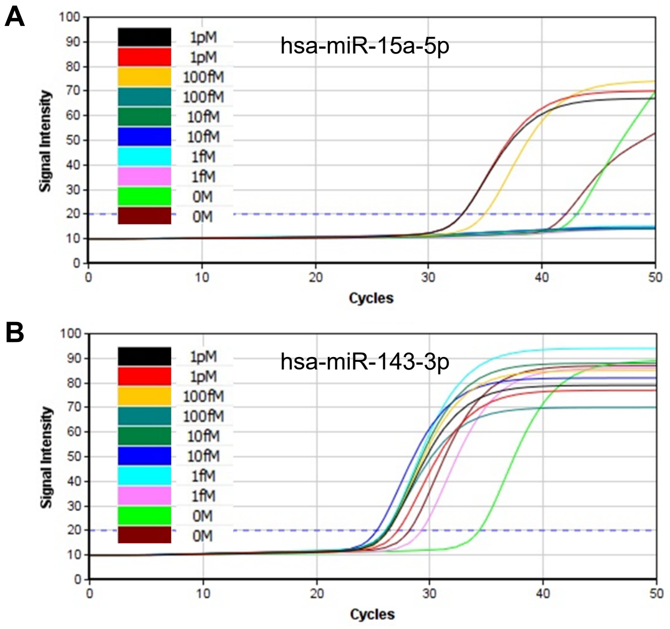

Figure S1: Thermal Cycle Profile of one-step real-time PCR for (A) hsa-miR-15a-5p and (B) hsa-miR-143-3p, measured after the RT.

were 50 and 45 °C for hsa-miR-15a-5p and hsa-miR-143-3p, respectively, and the elongation was conducted at 72 °C. The total runtime was approximately 30 min. We set the concentrations of the target miRNAs to be a range from 1 pM to 1 fM. The negative control was also included at 0 M.

In Figure S1A, detection profiles of hsa-miR-15a-5p are shown with colored curves, as indicated. It is verified that two profiles of 1 pM raise at 30 cycles, one of the profiles of 100 fM (yellow) start growing up at 33 cycles, two profiles of negative control (i.e., 0 M) grow up at approximately 41 cycles (green and brown), and others do not show their amplification. Thus, this one-step real-time RT-PCR technique failed to detect miRNAs with 100 fM or less concentrations; furthermore, the false positive reactions took place at 0 M. Although we repeated detections similar to Figure S1A, we did not observe any normal amplification showing that all the signals from 1 fM to 1 pM normally appeared and simultaneously that the false signal at 0 M was suppressed.

In Figure S1B, detection profiles of hsa-miR-143-3p are shown in a similar manner to that in Figure S1A. Obviously, signals rise at all the concentrations, which indicates that false positive reactions took place. This means that the one-step detection utterly failed.

Overall, we found that, depending on the target miRNA sequences and the primers, poorly

positive signals (Figure S1A) or all false signals (Figure S1B) appeared in the one-step real-time RT-PCR. Thus, this one-step technique, which is a typical one as one-step real-time RT-PCR techniques, is unreliable even at a rather high concentration of 1 pM.

S2. LAMP

As is discussed in the text, we tested RT-LAMP for miRNAs with following a procedure reported by Gao et al.[S1] In addition to the primers 1 and 2 noted in the text, forward-induced primer (FIP) and backward-induced primer (BIP) were designed along the concept by Gao et al. These sequences for hsa-miR-143-3p detection are listed in Table S1. The biotin labels represented with [Bio] were designed to immobilize the amplicons onto the metasurface FL biosensors.

Table S1: Sequences of FIP and BIP for hsa-miR143-3p, shown in the order from 5'-end (left) to 3'-end (right). Symbol [Bio] denotes biotin.

Species	Sequence
FIP	[Bio]TTTGCTGACGACTCCTTTTGTTGTCTGGAAGTGTGACGCGA
BIP	[Bio]TTTTAGCAGCACTGACTTTGTAATAGGACTGTCCGCCGCAC

Because of poor results in one-step and two-step RT-LAMPs, we conducted three-step RT-LAMP, which comprised the following reactions:

1. RT reaction using the primer 1 at 46 °C for 40 min to yield cDNA of the target miRNA. The reaction solution contained 1 μL primer 1 of 10 pmol, 0.2 μL ProtoScript II Reverse Transcriptase (M0368L, New England Biolabs), 1 μL 5×RT buffer, 0.5 μL 10×DTT, 1 μL dNTP mixture (2.5 millimolar (mM) each), and 1.4 μL target miRNA solution diluted with Nuclease-free water (314-09291, Nippon Gene).

2. PCR to use the primer 2 and to amplify the cDNA in a thermal steps, such as 98 °C for 30 s → (98 °C for 5 s → 45 °C for 30 s → 72 °C for 20 s)× 35 cycles. The PCR solution contained the 5 μL RT solutions and 20 μL PCR solution consisting of 2 μL of 20-pmol primer 2, 0.25 μL of 0.5-unit Phusion Hot Start Flex DNA Polymerase (M0535S, New England Biolabs), 5

μL 5×Phusion buffer, 2 μL dNTP mixture (2.5 mM each), 1.7 μL 50 mM $MgCl_2$, and 9.05 μL nuclease-free water (314-09291, Nippon Gene).

3. LAMP for the cDNA-amplified solution at 63 °C for 60 minutes, by adding the FIP and BIP in Table S1. We took 5 μL from the PCR reaction solution, added 20 μL LAMP solution composed of 2 μL of 20-pmol FIP, 2 μL of 20-pmol BIP and 12.5 μL 2×LAMP master mix (314-08951, Nippon Gene), and implemented the LAMP.

After this RT LAMP, the reacted solutions were cooled down to room temperature, the biotin- and FL-probes were added, and then hybridization with the probes was conducted in a sequence of (80 °C for 3 min → 51 °C for 30 min → room temperature).

Figure S2 shows typical results of the FL detections for the RT LAMP products, where the target miRNA was hsa-miR-143-3p and the concentrations were varied from 2 fM to 2 aM. We found that definite FL signals were observed only at 2 fM and that the FL signals at 200 aM and less were almost zero level or largely fluctuated. Even when going through these very elaborate procedures, we found that the miRNA at only 2 fM and higher concentrations can be detected and that this RT LAMP is unreliable at low concentrations in aM range. Overall, we could not reproduce the claim by Gao et al. that the 1 aM (i.e., 6 copies/test) detection was obtained.[S1]

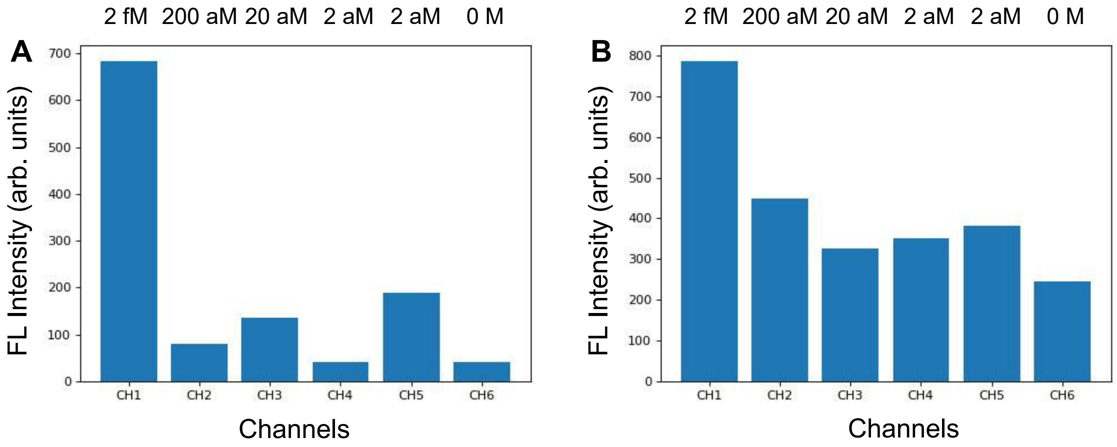

Figure S2: RT LAMP results. (A) and (B) Target miRNA was hsa-miR-143-3p, being detected in a range from 2 fM to 2 aM repeatedly.

Regarding the miRNA of hsa-miR-15a-5p, the RT-LAMP results were similar to those of hsa-

miR-143-3p in Figure S2. Although we do not repeat the results, we note that hsa-miR-15a5p at less than 2 fM was not detected successfully.

S3. Chromatography

We evaluated the two-step RT-PCR products (i.e., amplicons) using an efficient chromatography instrument (Labchip GX Touch 24) because we had interest in other potential techniques that can detect the RT-PCR products in a very efficient manner, similarly to the metasurface FL biosensors. Basically, the chromatography instrument conducts MF-chip-based time-of-flight measurement for DNAs including amplicons. We show a series of the chromatography results in Figure S3,S4, because of many panels. These were measured using a reagent kit for DNA detection (CLS760672, Revvity) and a MF chip (CLS138948, Revvity). The target miRNA, hsa-miR-143-3p, went through the two-step RT PCR, in which 48 PCR cycles were conducted. From Figure 3A in the text, we infer that the miRNAs at 5 aM are detectable under this condition.

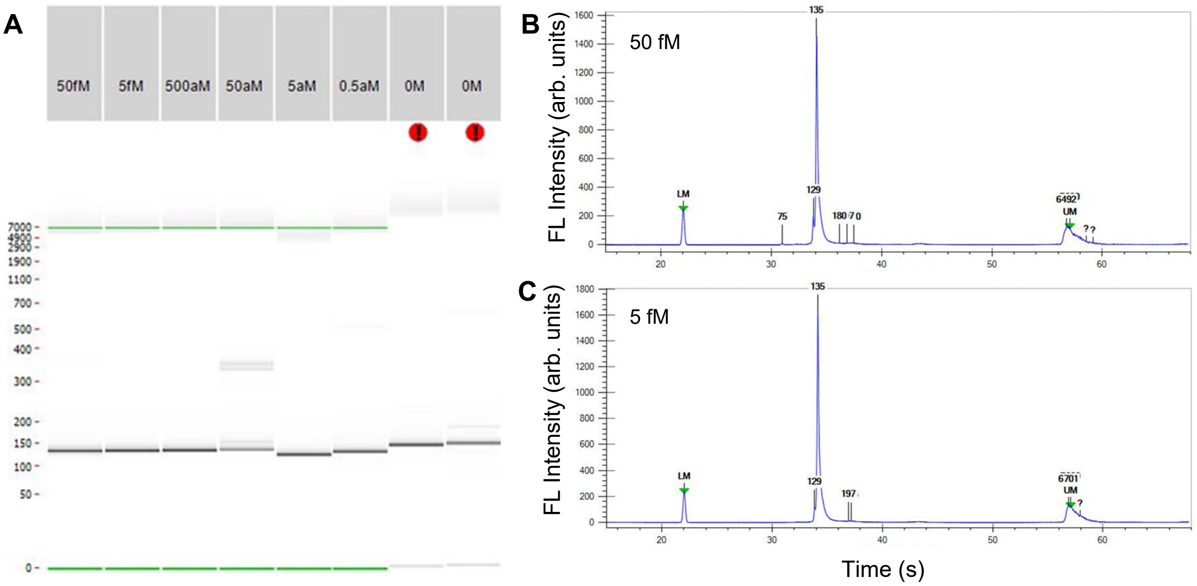

Figure S3: Chromatography results: part 1. (A) Detected molecular map, summing up a series of chromatography results. (B) and (C) Chromatography data, which are temporal profiles of time-of-flight FL signals at miRNA concentrations of 50 fM and 5 fM, respectively.

In Figure S3A, a summary map of a series of chromatography measurement is shown. The

amplicons are considered to have approximately 135 bases from the primer designs. Narrow bands appear around 135 bases at the miRNA concentrations from 50 fM to 0 M. This result is not a good signature because signals at 0 M implies false positive reactions.

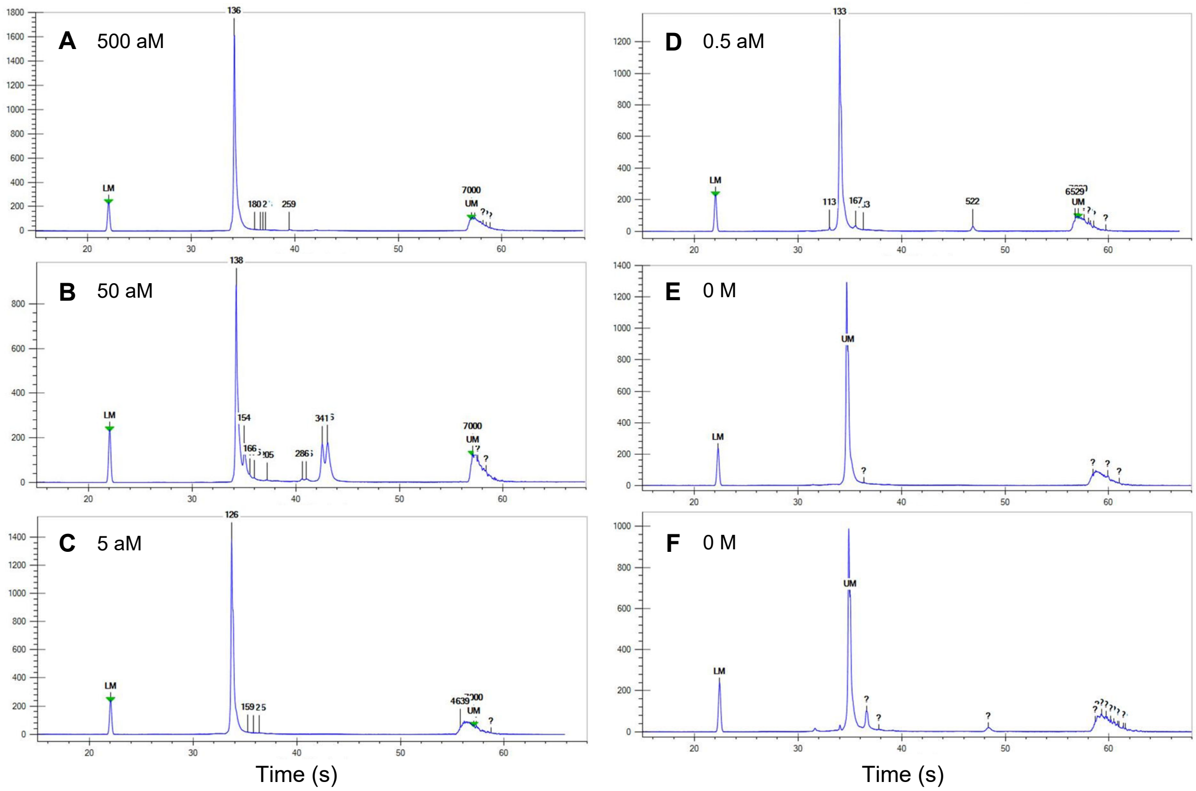

Figure S4: Chromatography results: part 2. (A)–(F) Target miRNA concentrations were 500, 50, 5, 0.5, 0, and 0 aM, respectively. All the vertical and horizontal axes commonly represent FL intensity and time, respectively.

Let us examine the measured data at each concentration, which are shown in Figure S3B,C and Figure S4. In the chromatography instrument, DNAs hybridize with FL probes in the DNA-specific kit and are detected via the FL signals. Calibrated lower-limit molecule (LM) and upper-limit molecule (UM) were also flowed together and enable to determine the lower and upper limits in the measurement. The LM and UM are indicated by green arrows in each panel, which correspond to 0 and 7,000 in Figure S3A. It is explicit that a distinct peak with a label of approximately 135 appears in each panel of Figure S3B,C and Figure S4, though auto-analysis application failed to correctly make labels at 0 M. This probably came from the broaden peak at the UM positions. Normally, the LM and UM peaks are isolated and sharp; however, each panel shows a broaden UM

peak, which made the analysis less precise. In addition to the broaden UM peak, we observed side bands around the peak with the label of approximately 135. These results strongly suggest that the RT-PCR products were smear; that is, unintended products were yielded and coexisted with the genuine amplicons.

The chromatography measurement and analysis are most likely to be unable to handle the amplicons in a selective manner with discriminating the genuine amplicons from the unintended products, whereas the metasurface FL biosensors work with the biotin- and FL-probes that are specific to the genuine amplicons (Figure 1C). Consequently, the metasurface biosensors properly function, even when the RT-PCR products include smear that is most likely to be mixed and/or aggregated amplified products.

S4. Reference Experiment on Cys-SA

Performance of the binding molecule, Cys-SA, on the metasurface FL biosensors was confirmed in a reference experiment. The configuration was simply with/without the Cys-SA. Without immobilizing the Cys-SA (that is, skipping the first step in Figure 1E), any biotin–streptavidin coupling does not take place on the metasurfaces, which is considered to result in substantially zero FL signal. Figure S5 shows a set of the results in this reference experiment.

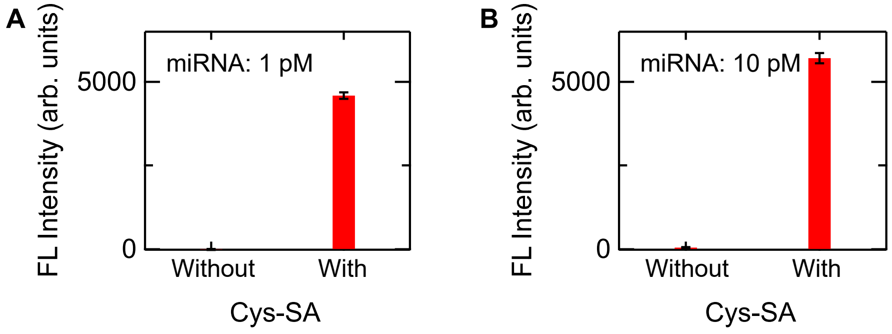

Figure S5: Capture effect by binding molecule, Cys-SA. (A) and (B) FL signals (red bars with error bars) on the metasurface biosensors, coming from the 1- and 10-pM target miRNA of hsa-miR-143-3p, respectively. Each panel includes the FL intensities with/without Cys-SA.

Irrespective of the target miRNA concentrations, it is obvious that measured FL intensities in

Figure S5 (red bars with error bars) were heavily reduced without the Cys-SA; practically, the FL intensities without Cys-SA were zero levels. These results substantiate that the Cys-SAs are certainly immobilized on the metasurfaces and efficiently function as binding molecules for the biotin-labeled amplicons.

The target miRNA, hsa-miR-143-3p, was gone through the two-step RT-PCR, whose procedure is described in the text (see also Figure 1B). The PCR cycles was set to 40. After the two-step RT-PCR, the amplicons were hybridized with the biotin and FL probes, as noted in the text (see also Figure 1C). We used multiple MF channels for each condition in Figure S5, and evaluated the averaged FL intensities (red bars) and standard deviations (error bars).

References

(S1) Gao, K.; Zhang, P.; Wang, H.; Wang, H.; Su, F.; Li, Z. Ultrasensitive homogeneous detection of microRNAs in a single cell with specifically designed exponential amplification. *Chem. Commun.* **2021**, *57*, 5570–5573.
